# Urolithin A-mediated augmentation of intestinal barrier function through elevated secretory mucin synthesis

**DOI:** 10.1038/s41598-024-65791-x

**Published:** 2024-07-08

**Authors:** Takeshi Yasuda, Tomohisa Takagi, Kohei Asaeda, Hikaru Hashimoto, Mariko Kajiwara, Yuka Azuma, Hiroaki Kitae, Yasuko Hirai, Katsura Mizushima, Toshifumi Doi, Ken Inoue, Osamu Dohi, Naohisa Yoshida, Kazuhiko Uchiyama, Takeshi Ishikawa, Hideyuki Konishi, Yuichi Ukawa, Akiko Kohara, Masatake Kudoh, Ryo Inoue, Yuji Naito, Yoshito Itoh

**Affiliations:** 1 Molecular Gastroenterology and Hepatology, Graduate School of Medical Science, Kyoto Prefectural University of Medicine, Kyoto, 602-8566 Japan; 2 Department for Medical Innovation and Translational Medical Science, Graduate School of Medical Science, Kyoto Prefectural University of Medicine, Kyoto, 602-8566 Japan; 3 Department of Human Immunology and Nutrition Science, Graduate School of Medical Science, Kyoto Prefectural University of Medicine, Kyoto, 602-8566 Japan; 4 Daicel Corporation, Healthcare SBU, Tokyo, 108-8230 Japan; 5 Daicel Corporation, Healthcare SBU, Niigata, 944-8550 Japan; 6 Laboratory of Animal Science, Department of Applied Biological Sciences, Faculty of Agriculture, Setsunan University, Hirakata, 572-8508 Japan

**Keywords:** Urolithin A, Mucin 2 production, AhR signaling, Nrf2 pathway, Intestinal barrier function, Colitis prevention, Cell biology, Gastroenterology

## Abstract

Maintaining the mucus layer is crucial for the innate immune system. Urolithin A (Uro A) is a gut microbiota-derived metabolite; however, its effect on mucin production as a physical barrier remains unclear. This study aimed to elucidate the protective effects of Uro A on mucin production in the colon. In vivo experiments employing wild-type mice, NF-E2-related factor 2 (Nrf2)-deficient mice, and wild-type mice treated with an aryl hydrocarbon receptor (AhR) antagonist were conducted to investigate the physiological role of Uro A. Additionally, in vitro assays using mucin-producing cells (LS174T) were conducted to assess mucus production following Uro A treatment. We found that Uro A thickened murine colonic mucus via enhanced mucin 2 expression facilitated by Nrf2 and AhR signaling without altering tight junctions. Uro A reduced mucosal permeability in fluorescein isothiocyanate-dextran experiments and alleviated dextran sulfate sodium-induced colitis. Uro A treatment increased short-chain fatty acid-producing bacteria and propionic acid concentration. LS174T cell studies confirmed that Uro A promotes mucus production through the AhR and Nrf2 pathways. In conclusion, the enhanced intestinal mucus secretion induced by Uro A is mediated through the actions of Nrf-2 and AhR, which help maintain intestinal barrier function.

## Introduction

Urolithin A (Uro A) is an intestinal metabolite produced by the gut microbiota from ellagic acid, a polyphenol found in pomegranates, nuts, and several berries^1,2^. Polyphenols function mainly in the intestines^3^. Less than 10% of ingested polyphenols are absorbed through metabolism, with the remaining 90% persisting in the intestine for an extended period^3^. Through this process, polyphenols suppress the onset of inflammation in the gastrointestinal mucosa caused by oxidative stress^4^ and prevent obesity by inhibiting lipid absorption^5^.

Among polyphenols, ellagic acid possesses anti-inflammatory and antioxidant effects^6^. Dietary supplementation with pomegranate extract containing ellagic acid decreases oxidative stress in the colonic mucosa and reduces the colitis in an interleukin-10-deficient mouse model^7,8^. Recently, various polyphenols have been speculated to play specific roles in the gastrointestinal tract, particularly in relation to the intestinal bacteria in the colon^9,10^. Clinical trials also support this result, showing that a polyphenol-rich diet in elderly patients with a leaky gut can alter intestinal microbiota and improve intestinal permeability^11^.

From the viewpoint of suppressing intestinal permeability, mucosal surface barriers are divided into physical and chemical barriers^12^. Physical barriers include tight junctions between epithelial cells, mucin layers, and glycocalyx, whereas chemical barriers include antimicrobial peptides and secretory IgA antibodies. Regarding tight junctions, Uro A reportedly activates the aryl hydrocarbon receptor (AhR) and NF-E2-related factor 2 (Nrf2)-dependent pathway and upregulates epithelial tight junction proteins^13^. However, the mucin layers on the intestinal surface are also essential for barrier function^14–16^. Approximately 21 known mucin genes have been identified in human tissues, and mucin 2 (MUC2) is the main component of secretory mucins in the colon^17–19^. In the colon, the inner mucus layer separates the gut microbiota and epithelial layer, which prevents excessive immune response against the host intestinal bacteria^20^. In MUC2-deficient mice, the inner mucus layer does not form, allowing intestinal bacteria to invade the colonic mucosa and cause colitis^21–23^. Furthermore, reduced mucosal layer thickness increases susceptibility to intestinal inflammation by causing dysbiosis. For example, in NLRP6-deficient mice, mucus release from goblet cells is impaired, resulting in a partially formed inner mucus layer, which in turn causes dysbiosis and increased susceptibility to dextran sulfate sodium (DSS)-induced colitis^24^. As aforementioned, the benefit of Uro A for tight junctions has already been elucidated; however, the effect of Uro A on mucin production as a physical barrier remains unclear. Thus, this study aimed to investigate the impact of Uro A on the intestinal barrier function via mucus production.

## Results

### Uro A increased the mucus layer thickness

**Figure 1:**
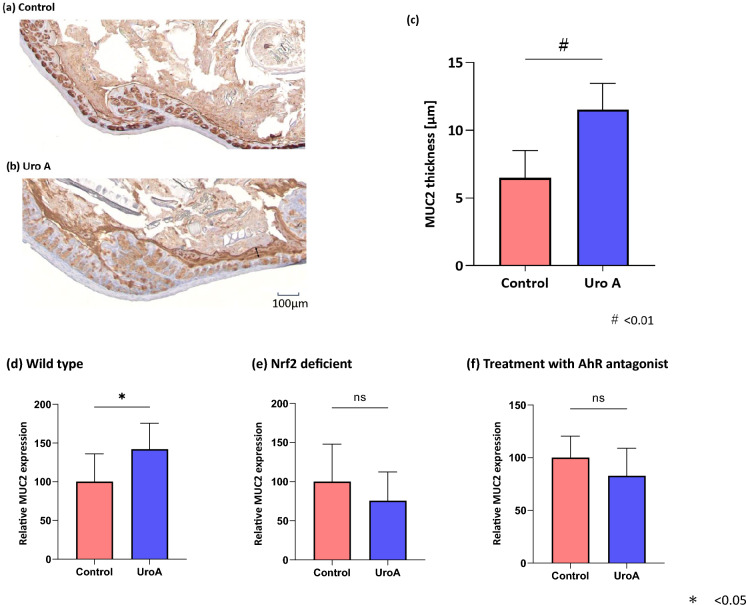
Results of the administration of Uro A to the 6-week mice. (**a**) Control group: The MUC2 immunostaining of wild-type (C57BL/6) mice colon. The MUC2-positive mucus layer is dark brown. (**b**) Uro A group: The MUC2 immunostaining of the colon in wild-type (C57BL/6) mice treated with Uro A (100 mg/kg). The MUC2-positive mucus layer is stained dark brown (marked by "↔"). (**c**) The thickness of the MUC2-positive mucus layer in the Uro A group is significantly greater than that in the control group (n = 6, control group; 6.47 ± 2.03 µm, Uro A group; 11.52 ± 1.93 µm, p < 0.01, Wilcoxon rank sum test). (**d**) As evaluated by ELISA, the MUC2 protein level in the colonic mucosal epithelium is significantly higher in the Uro A group than in the control group (n = 6, control group; 100.0 ± 35.8, Uro A group; 142.0 ± 33.6, p = 0.041, Wilcoxon rank sum test). (**e**) Uro A treatment does not increase MUC2 protein levels in the colonic epithelium of Nrf2-deficient mice (n = 6, control group; 100.0 ± 48.0, Uro A group; 75.6 ± 36.8, p = 0.309, Wilcoxon rank sum test). (**f**) The MUC2 protein level in mice treated intraperitoneally with an AhR antagonist did not increase in the colonic epithelium after Uro A treatment (n = 6, control group; 100.0 ± 20.3, Uro A group; 82.8 ± 26.1, p = 0.222, Wilcoxon rank sum test). Uro A, urolithin A; MUC2, mucin 2; ELISA, enzyme-linked immunosorbent assay; Nrf2, NF-E2-related factor 2.

The mice were divided into two groups: (a) wild-type (WT, C57BL/6) mice (control group) and (b) WT (C57BL/6) mice treated with Uro A (Uro A group). Cross-sections of the collected colon were stained with MUC2, the MUC2-positive mucus layer length was measured at nine different sites in each group, and the mean value was determined. Immunostaining for MUC2 showed that the thickness of the MUC2-positive mucus layer in the Uro A group significantly increased compared with that in the control group (control group; 6.47 ± 2.03 µm, Uro A group; 11.52 ± 1.93 µm, p < 0.01, Fig. 1a–c).

### Uro A increased the MUC2 protein level in the mucosal epithelium

MUC2 protein levels in the colonic mucosal epithelium were significantly higher in the Uro A group than that in the control group (control group; 100.0 ± 35.8, Uro A group; 142.0 ± 33.6, p = 0.041; Fig. 1d). In Nrf2-deficient mice, Uro A treatment did not increase MUC2 protein levels in the colonic epithelium (control group; 100.0 ± 48.0, Uro A group; 75.6 ± 36.8, p = 0.309, Wilcoxon rank sum test, Fig. 1e). Similarly, in mice treated intraperitoneally with an AhR antagonist, MUC2 protein levels did not increase in the colonic epithelium after treatment with Uro A (control group; 100.0 ± 20.3, Uro A group; 82.8 ± 26.1, p = 0.222, Fig. 1f).

### In vitro study of MUC2 expression by the stimulation with Uro A

**Figure 2:**
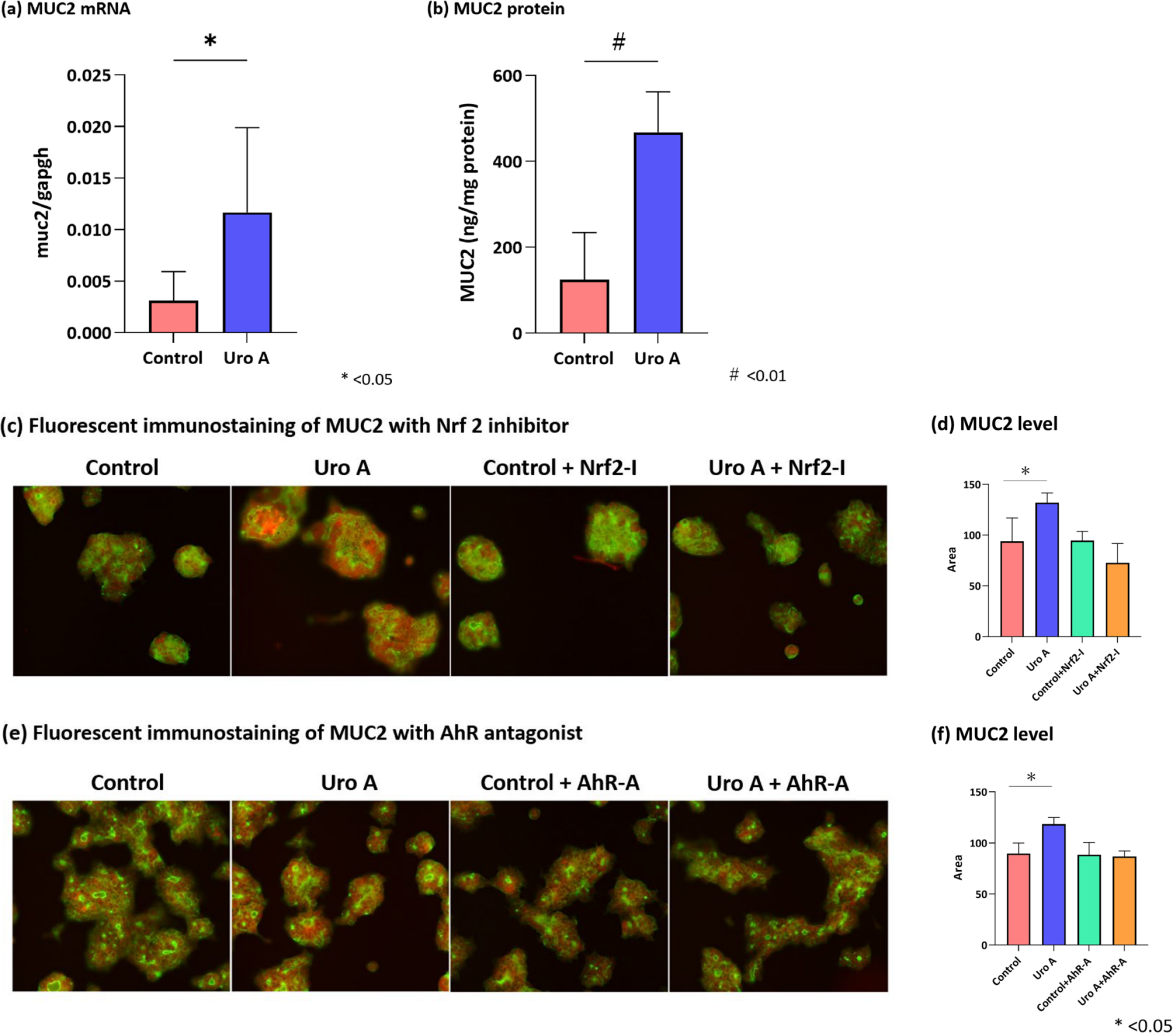
Results of the in vitro study using LS174T cells (colon mucus cells). (**a**) MUC2 mRNA levels in LS174T cells increased significantly after Uro A administration (control group; 0.003 ± 0.003, Uro A group; 0.012 ± 0.008, p = 0.02, Wilcoxon rank sum test). (**b**) MUC2 protein levels in LS174T cells significantly increased after Uro A administration (control group; 125.0 ± 109.4 ng/mg protein, Uro A group; 467.8 ± 93.9 ng/mg protein, p < 0.01, Wilcoxon rank sum test). (**c**, **d**) Fluorescence immunostaining of LS174T cells. TRITC was used for MUC2 (red), and GFP was used for the cell membrane (green). A comparison of the control, Uro A, control with ML 385 (Nrf 2 inhibitor), and Uro A with ML 385 was performed. ImageJ analysis of fluorescence immunostaining shows that the intensity of MUC2 fluorescence expression in LS174T cells after treatment with Uro A is significantly higher than that in the control group (control group; 93.9 ± 22.9, Uro A group; 131.9 ± 9.7, p = 0.030, Wilcoxon rank sum test). Additionally, MUC2 fluorescence did not increase after treatment with ML 385 (control group; 94.7 ± 9.0, Uro A group; 72.6 ± 19.2, p = 0.194, Wilcoxon rank sum test). (**e**, **f**) Fluorescence immunostaining of MUC2 with control, Uro A, control with AhR antagonist, and Uro A with AhR antagonist shows that the strength of MUC2 fluorescence in LS174T cells after treatment with Uro A and AhR antagonists did not increase after treatment with CH223191 (AhR antagonist, control group; 88.3 ± 12.3, Uro A group; 86.7 ± 5.4, p = 1.000, Wilcoxon rank sum test). Uro A, urolithin A; mRNA, messenger RNA; MUC2, mucin 2; TRITC, tetramethylrhodamine isothiocyanate; GFP, green fluorescent protein.

To ascertain the upregulation of MUC2 stimulated by Uro A, LS174T cells were treated with Uro A. MUC2 mRNA (control group; 0.003 ± 0.003, Uro A group; 0.012 ± 0.008, p = 0.02) and MUC2 (control group; 125.0 ± 109.4 ng/mg protein, Uro A group; 467.8 ± 93.9 ng/mg protein, p < 0.01) protein levels in LS174T cells increased significantly after Uro A administration (Fig. 2a,b).

Fluorescence immunostaining showed that the strength of MUC2 fluorescence (recognized as a reddish color) in LS174T cells after treatment with Uro A was significantly higher than that in the control group (control group; 93.9 ± 22.9, Uro A group; 131.9 ± 9.7, p = 0.030). In addition, MUC2 fluorescence expression did not increase after treatment with ML 385 (Nrf2 inhibitor, control group; 94.7 ± 9.0, Uro A group; 72.6 ± 19.2, p = 0.194) (Fig. 2c,d). Similarly, MUC2 fluorescence did not increase after treatment with CH223191 (AhR antagonist, control group; 88.3 ± 12.3, Uro A group; 86.7 ± 5.4, p = 1.000) (Fig. 2e,f).

### Uro A administration and colonic mucosal permeability

**Figure 3:**
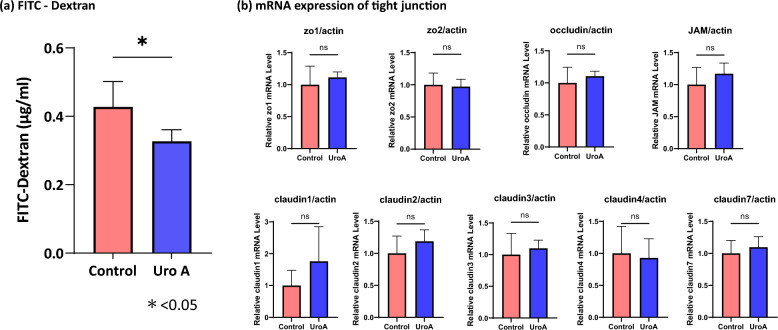
Alternation of colon permeability after administration of Uro A. FITC–Dextran was used to investigate the alternation of colon permeability. (**a**) The concentration of FITC-dextran (4 kDa) in the portal vein shows that the plasma FITC-dextran level in the Uro A group is significantly lower than that in the control group (n = 6, control group; 0.43 ± 0.07 µg/mL, Uro A group; 0.33 ± 0.03 µg/mL, p = 0.013, Wilcoxon rank sum test). (**b**) The mRNA expression of tight junction proteins in the colon epithelium shows that the levels of zo-1, zo-2, occludin, JAM, and claudin 1, 2, 3, 4, and 7 do not differ significantly between the Uro A and control groups (n = 6, zo1: control group; 1.00 ± 0.12, Uro A group; 1.11 ± 0.03, p = 0.68, zo2: control group; 1.00 ± 0.07, Uro A group; 0.97 ± 0.05, p = 0.53, occludin: control group; 1.00 ± 0.10, Uro A group; 1.10 ± 0.03, p = 0.68, JAM: control group; 1.00 ± 0.11, Uro A group; 1.17 ± 0.07, p = 0.30, claudin1: control group; 1.00 ± 0.19, Uro A group; 1.76 ± 0.44, p = 0.30, claudin2: control group; 1.00 ± 0.11, Uro A group; 1.19 ± 0.07, p = 0.40, claudin3: control group; 1.00 ± 0.13, Uro A group; 1.10 ± 0.05, p = 0.40, claudin4: control group; 1.00 ± 0.17, Uro A group; 0.93 ± 0.12, p = 1.00, claudin7: control group; 1.00 ± 0.07, Uro A group; 1.10 ± 0.06, p = 0.68, Wilcoxon rank sum test). Uro A, urolithin A; FITC, fluorescein isothiocyanate; mRNA, messenger RNA.

Fluorescein isothiocyanate (FITC)-dextran was used to investigate alterations in colon permeability. The results showed that the plasma FITC-dextran level in the Uro A group was significantly lower than that in the control group (control group; 0.43 ± 0.07 µg/mL, Uro A group; 0.33 ± 0.03 µg/mL, p = 0.013) (Fig. 3a).

Since previous reports have demonstrated that Uro A enhances tight junction^13^, we evaluated the mRNA expression of tight junction proteins. The mRNA expression of zo-1, zo-2, occludin, JAM, and claudin 1, 2, 3, 4, and 7 did not differ significantly between the Uro A and control groups (Fig. 3b).

### Effect of Uro A on intestinal microbiota

**Figure 4:**
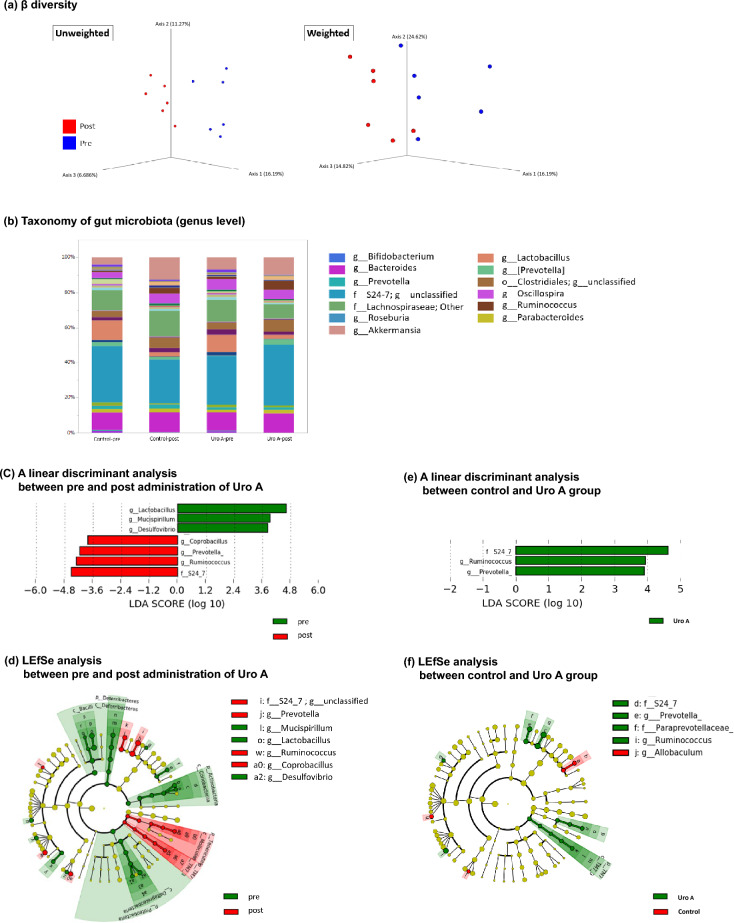
Data for analysis of intestinal bacteria in fresh mouse stool. (**a**) The result of ß diversity shows that the gut microbiota composition differed significantly between the control and the Uro A group in both unweighted and weighted analyses (p < 0.01, p < 0.01, respectively. PERMANOVA). (**b**) Taxonomy of gut microbiota at the genus level shows that the predominant five genera in both groups before the administration of Uro A included unclassified genus belonging to the family *S24-7*, genera belonging to the family *Lachnospiraceae*, genera *Bacteroides*, *Akkermansia*, and *Lactobacillus*. (**c**, **d**) An LDA ((log10) > 3.5) and LEfSe analysis after administration of Uro A in wild-type mice. A difference in the alternation of several genera is observed after the administration of Uro A. In the Uro A group, the abundance of the unclassified genus belonging to the family *S24-7* (before: 27.7%, after: 34.9%, p = 0.045, Wilcoxon signed-rank test), genus *Ruminococcus* (before: 1.3%, after: 5.0%, p < 0.01, Wilcoxon signed-rank test), and genus *Prevotella* (before: 0.2%, after: 3.1%, p < 0.01, Wilcoxon signed-rank test) significantly increased after 1 week of oral Uro A administration. (**e**, **f**) An LDA ([log10] > 3.5) and LEfSe analysis between the control and Uro A groups after Uro A administration. In the Uro A group, the abundance of the unclassified genus belonging to the family *S24-7* (control group: 25.0%, Uro A group: 34.9%, p = 0.013, Wilcoxon signed-rank test), genus *Ruminococcus* (control group: 3.4%, Uro A group: 5.0%, p = 0.045, Wilcoxon signed-rank test), and genus *Prevotella* (control group: 1.4%, Uro A group: 3.1%, p = 0.031, Wilcoxon signed-rank test) significantly increased. Uro A, urolithin A; LDA, linear discriminant analysis; LEfSe, linear discriminant analysis effect size.

WT mice were treated with Uro A for 1 week to examine changes in the diversity and abundance of the gut microbiota before and after administration. Additionally, the feces of WT mice after Uro A administration were compared with those of the control group. Principal coordinate analysis showed that the gut microbiota composition differed significantly after Uro A administration in the unweighted and weighted analyses (p < 0.01) (Fig. 4a). The taxonomy of the gut microbiota at the genus level revealed that, in the Uro A group, the abundance of short-chain fatty acid (SCFA)-producing bacteria, such as family *S24-7*, genus *Ruminococcus*, and genus *Prevotella*, significantly increased after 1 week of administration of oral Uro A (Fig. 4b–d). Compared to the control group, the abundance of three genera significantly increased in the Uro A group after 1 week of administration of oral Uro A (Fig. 4b–f).

### Effect of Uro A on fecal SCFA levels

A comparison of SCFA concentrations in stool before and after Uro A administration showed that propionate was significantly elevated after Uro A administration. Other SCFAs showed no significant differences before and after the administration of Uro A (Table 1).

**Table 1:** Concentration of short-chain fatty acids in fresh stool.

SCFA	Before (μmol/g)	After (μmol/g)	P value
Acetate	44.16 ± 15.46	52.58 ± 34.13	0.689
Propionate	1.76 ± 1.01	4.03 ± 3.14	0.045
Butyrate	0.57 ± 0.48	0.78 ± 0.64	0.471
Lactate	0.07 ± 0.06	0.17 ± 0.19	0.23
Formate	1.29 ± 0.37	1.17 ± 0.82	0.81
Succinate	0.53 ± 0.15	0.52 ± 0.36	1

### Effect of Uro A on the colonic damage induced by DSS administration

**Figure 5:**
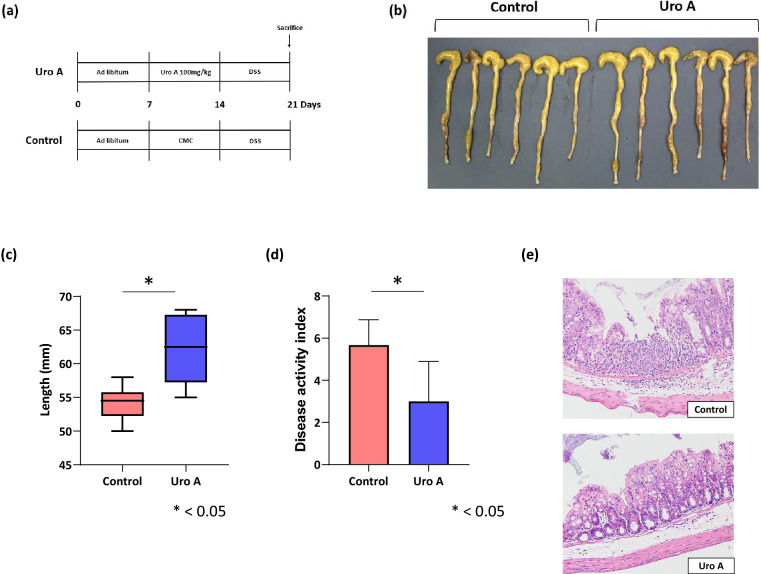
Schematic diagram of the induction of the DSS colitis mouse model. (**a**) After keeping ad libitum feeding for 1 week, Uro A 100 mg/kg was administered orally twice daily for male wild-type mice. Subsequently, DSS was administered through the drinking water for 7 days. The mice were euthanized under anesthesia 7 days after initiating DSS treatment. (**b**, **c**) The entire colon was resected from the cecum to the anus, and the colon length was measured as an indirect marker of intestinal inflammation. Significant shortening of the intestinal length is observed in the control group (control group (n = 6); 54.2 ± 2.6 mm, Uro A group (n = 6); 62.2 ± 5.8 mm, p = 0.018, Wilcoxon rank sum test). (**d**) Effects of Uro A administration on the DAI. The Uro A group show a markedly reduced DAI after 1 week of DSS administration (control group (n = 6); 5.7 ± 1.2, Uro A group (n = 6); 3.0 ± 1.9, p = 0.033, Wilcoxon rank sum test). (**e**) Hematoxylin–eosin staining of tissue sections shows marked inflammatory cell infiltration in the mucosa and submucosa with destruction of gland duct structures in the control group. In contrast, inflammatory cell infiltration and destruction of gland duct structures are mild in the Uro A group. DSS, dextran sulfate sodium; Uro A, urolithin A, DAI, disease activity index.

After exposure to DSS, we evaluated the colon length and disease activity index (DAI). Significant shortening of the intestinal length was observed in the control group (control group; 54.2 ± 2.6 mm, Uro A group; 62.2 ± 5.8 mm, p = 0.018) (Fig. 5b,c). In the Uro A group, the DAI significantly lower in comparison with control group 1 week after DSS administration (control group; 5.7 ± 1.2, Uro A group; 3.0 ± 1.9, p = 0.033) (Fig. 5d). Hematoxylin and eosin staining of tissue sections showed significant inflammatory cell infiltration in the mucosa and submucosa with destruction of gland duct structures in the control group. In contrast, inflammatory cell infiltration and destruction of gland duct structures were mild in the Uro A group (Fig. 5e).

### Effect of Uro A on the colonic damage induced by TNBS administration

After exposure to TNBS, mice exhibited severe colitis in the colon, as indicated by hyperemia, edema, wall hypertrophy, and ulcerative lesions, reflected in the macroscopic score. Uro A treatment reduced the macroscopic score after TNBS administration (p = 0.077) (Fig. S2a–c).

## Discussion

We investigated the role of Uro A in gut barrier function. We found that stimulation with Uro A increased MUC2 protein levels in colonic epithelial cells through the AhR and Nrf2 pathways. This is the first study to demonstrate that Uro A intake enhances intestinal barrier function by increasing the mucus layer thickness of the colonic epithelium through MUC2 production.

In the present study, experiments in a mouse model showed that Uro A administration increased the amount of MUC2 in the colon epithelium and the thickness of MUC2-positive mucus in the mouse colonic epithelium. As reported previously, an increase in intracellular MUC2 increases MUC2 secretion^14^. Furthermore, evaluation using 4 kDka FITC-dextran showed that intestinal permeability was suppressed in the Uro A-treated group. Although previous studies have demonstrated that Uro A enhances barrier function by upregulating tight junction proteins^13^, our study could not show a significant difference in tight junction protein expression. Hence, our results showed that other than upregulating the expression of tight junction proteins in the epithelium as reported previously, increased intestinal mucus stimulated by Uro A suppresses leaky gut physically. Notably, these changes were not observed in Nrf2-deficient mice or mice treated intraperitoneally with an AhR antagonist. Experiments using human colon epithelial cells also showed that MUC2 in LS174T cells was upregulated by Uro A. However, this phenomenon was not observed after treatment with an Nrf2 inhibitor or an AhR antagonist.

Both the in vivo and in vitro findings suggest that the effects of Uro A on MUC2 production occur, at least in part, through the Nrf2 and AhR pathways. The signaling pathway through which Uro A acts on MUC2 expression has not been reported in detail yet. However, the relationship between AhR signaling and intestinal barrier function has been investigated recently. For example, a study using a mouse model reported that activation of AhR signaling promotes goblet cell differentiation and enhances MUC2 expression^25^. Furthermore, a high-fat diet decreases AhR signaling, which decreases MUC2 expression^26^. Regarding Nrf2, a study using low molecular seleno-aminopolysaccharide (LSA), which increases goblet cells and mucus, reported that LSA downregulates Keap1 expression and upregulates Nrf2 expression, thereby reducing abnormal changes in MUC2 and increasing the mucus layer in the intestine^27^. These previous reports indicate that the dioxin receptor AhR and the transcription factor Nrf2 are associated with MUC2 expression, thus supporting our results.

To replicate the findings by Singh et al.^13^, we examined the effect of Uro A in the mouse DSS and TNBS colitis models. In our DSS and TNBS colitis mouse model, mice treated with Uro A for 1 week exhibited less colonic damage compared to those in the control group, consistent with previous reports. Another report also showed that DSS enteritis was exacerbated in a mouse model treated with a drug that decreases intestinal mucus^28^. Furthermore, another report using a DSS colitis mouse model indicated that medication sustaining the number of goblet cells led to the maintenance of MUC2 mucus and the amelioration of DSS colitis^29^. Hence, the amount of intestinal mucus is crucial for protection against colitis. Our results showed that, similar to the materials used in previous reports, Uro A alleviated DSS and TNBS colitis in mice. In addition to the mouse colitis model, inflammatory bowel disease, which has recently been on the rise worldwide, is caused by abnormalities in the intestinal environment, mucosal barrier, and immune system^30^. Our results also imply that Uro A can potentially be a new inflammatory treatment by targeting the mucosal barrier.

Regarding the changes in the gut microbiota, our study revealed that the examination of the stools of mice before and after Uro A treatment showed a significant change in the β-diversity. The unclassified genus belonging to the family *S24-7*, genus *Ruminococcus*, and genus *Prevotella* significantly increased at the genus level. Notably, all these bacteria were SCFA-producing^31–33^. Examination of SCFA concentrations in stools in our study showed that propionic acid levels significantly increased after Uro A administration. Family *S24-7* increases with the long-term consumption of exopolysaccharides in fermented foods. In gnotobiotic mice that were colonized with the *S24-7* group, exopolysaccharide intake markedly enhanced propionic acid production among SCFAs^34^. Kajiwara et al. found that partially hydrolyzed guar gum (PHGG) administration increased MUC2 production in the mouse intestine. Notably, they also reported that PHGG administration increased the abundance of family *S24-7* and SCFA concentration, including propionic acid, in the intestine, consistent with our findings regarding Uro A administration^35^. Propionic acid affects intestinal immune function via the G protein-coupled receptor (GPR)41, and GPR43 inhibits local neutrophil migration in the colon and regulates colonic Treg cell homeostasis^36,37^. Regarding mucus production, a previous report using the same LS174T cells as our study argued that among SCFAs, succinic acid induces MUC2 expression via the AKT pathway, and propionic acid did not upregulate MUC2^35^. These results suggest that the stimulatory effect of UroA on intestinal mucus secretion does not operate directly via propionic acid produced by gut microbiota. However, Ma et al.^38^ revealed that propionic acid in goblet cells is metabolized via a β-oxidation-like pathway and induces hypoxia. Subsequently, specific activation of HIF-2α, which promotes MUC2 expression in LS174T goblet cells, increases mucus production and contributes to enhance the intestinal mucosal barrier. Although we did not study the induction of HIF-2α, this β-oxidation-like pathway may have been a factor involved in our results.

The current study had some limitations. First, the study did not confirm the functionality of the AhR antagonist CH-223191 and Nrf2 inhibitor ML385 at the concentration used in our test system. Second, it could not clarify the direct signal pathway from Nrf2/AhR to Muc2 expression. This is because the Muc2 production pathway is complex and there have been no reports of it directly targeting the AhR or Nrf2 genes. Regulation of intestinal mucus involves a variety of factors such as synthesis and secretion, and gene expression is not necessarily reliable. Therefore, in the present study immunostaining and protein expression were used to evaluate the results. Third, the study did not prove a causal link between mucin production and prevention of dysbiosis upon Uro A administration. Fourth, in contrast to previous reports^13^, the expression of tight junction mRNA was not elevated in the current study; however, the mechanism of this discrepancy could not be elucidated.

In conclusion, our results suggest that the MUC2-producing effect of Uro A affects the colon via AhR- and Nrf2-dependent pathways and is involved in the maintenance of the mucosal barrier in accordance with changes in the intestinal microbiota and SCFA.

## Materials and methods

### Study sample

Six-week-old male WT mice (C57BL/6) were purchased from Shimizu Laboratory Supplies (Kyoto, Japan). Similarly, six-week-old male Nrf2-deficient mice were purchased from the RIKEN Bioresource Center through the National BioResource Project (Ibaraki, Japan). The mice were caged individually in a room maintained at 18–24 °C with 40–70% relative humidity and a 12-h light/dark cycle. Mice had unrestricted access to food and potable water and were fed the rodent diet CE-2 (Nihon Clea, Tokyo, Japan) for 7 days during acclimatization.

### Reagents

All chemicals were prepared immediately before use. Uro A was generously provided by the Daicel Corporation (Osaka, Japan). The AhR antagonist CH-223191 and Nrf2 inhibitor ML385 were purchased from Selleck Biotech (Houston, TX, USA). All other chemicals were of the highest commercially available quality.

### Mouse model in this study

Male WT mice were divided into the control and Uro A-treated groups. Uro A, dissolved in carboxymethyl cellulose (CMC) at a dosage of 20 mg/kg or 100 mg/kg, was administered orally twice daily for 7 days as an exploratory experiment. In the control group, CMC was administered orally twice daily for 7 days. mRNA expression analysis of MUC2 in the colon epithelium indicated a gradual increase in MUC2 levels in the control, 20 mg/kg Uro A, and 100 mg/kg Uro A group (Fig. S1). To elucidate the mechanism by which Uro A produces mucus, we used the 100 mg/kg dose, which was more effective, although the previous report used a 20 mg/kg dose. We also included Nrf2-deficient (6 weeks old) and WT (C57BL/6) mice treated with an AhR antagonist (six mice per group). For AhR antagonism, CH-223191 was dissolved in corn oil, and 10 mg/kg was administered intraperitoneally 24 h before Uro A administration^39^.

### Analysis of the mucus layer thickness in the *colon*

The collected colon tissues were fixed in Karnois solution for 3 h and immersed in ethanol. The anti-MUC2 antibody was used to stain the colon sections to highlight the MUC2 expression. Measurements of MUC2 thickness were conducted at nine distinct sites within each experimental group at 40× magnification. The computed averages of these measurements constituted the data used in this study.

### Analysis of the MUC2 production level in the *colon*

Using the methodology described by Ohkawa et al.^40^, MUC2 was quantified in the colonic mucosa as a marker for mucin production. After the experimental procedures, the colon mucosa (4 cm length of the distal colon) was scraped off using two glass slides, followed by homogenization in 1.5 mL of 10-mmol/L potassium phosphate buffer (pH 7.8) containing 30 mmol/L KCl using a Teflon Potter–Elvehjem homogenizer. The MUC2 concentration within the tissue homogenates was measured using the SEA705Mu Mucin enzyme-linked immunosorbent assay (ELISA) kit model (Cloud-Clone Corp. Wuhan, China), in strict accordance with the manufacturer’s protocol. For MUC2 concentrations, each control group was settled as 100.

### Evaluation of intestinal mucosa permeability

FITC-dextran (Krackeler Scientific Inc., Albany, NY) was used to investigate the alterations in colon permeability. A dose of 4 kDa FITC-dextran (10 mg/0.25 mL/mouse) was orally administered, and plasma samples were collected from the portal vein 3 h post-administration^41,42^. FITC-dextran is a valuable tool for assessing intestinal permeability, especially because it remains in the colon until excreted, with 4 kDa FITC-dextran being the optimal molecular weight^43,44^.

### Messenger RNA (mRNA) analysis

The expression of MUC2 and tight junction proteins was determined using quantitative reverse transcription-polymerase chain reaction (qRT-PCR). Total RNA was isolated from murine colonic mucosa using the acid guanidinium phenol–chloroform technique with TRIzol Reagent according to the manufacturer’s guidelines. The RNA concentration was determined using the 260 and 280 nm absorbance value ratio. The extracted RNA samples were stored at − 80 °C until required. RNA was reverse transcribed to generate complementary DNA (cDNA) using the High Capacity cDNA Reverse Transcription Kit (Applied Biosystems). Subsequently, the cDNA was used for qRT-PCR. The PCR primers used to detect MUC2; claudin-1, -2, -3, -4, and-7; occludin; JAM-A; ZO-1; and ZO-2 are listed in Table 2. qRT-PCR was performed using Power SYBR Green PCR Master Mix and a real-time PCR system (model 7300; Applied Biosystems, Foster City, CA, USA). The PCR protocol comprised 40 cycles of denaturation at 95 °C for 15 s, annealing of primers at 60 °C for 1 min, followed by a melting curve analysis involving a temperature ramp from 60 to 95 °C. Gene expression quantification was calculated with respect to the reference genes β-actin.

**Table 2:** qRT-polymerase chain reaction primers.

Gene	Forward primer	Reverse primer
*MUC 2*	5-TGGGTGTCCTCGTCTCCTACA-3′	5′-TGTTGCCAAACCGGTGGTA-3′
*Claudin-1*	5′-TGACCGCTCAGGCCATCTAC-3′	5′-CTGCCCGGTGCTTTGC-3′
*Claudin-2*	5′-CCTCCCTTGGCGTCCAA-3′	5′-GTGCCTAACAGCCCCAAAAG-3′
*Claudin-3*	5′-TCATCACGGCGCAGATCA-3′	5′-CTCTGCACCACGCAGTTCA-3′
*Claudin-4*	5′-TGTCCTGGACCGCTCACAA-3′	5′-CCCGGAAGCCACCATAGG-3′
*Claudin-7*	5′-GCGCGTCCCGTCTTTTCT-3′	5′-CAGTTGCAGGCCCGAGTT-3′
*Occludin*	5′-AGCCTCGGTACAGCAGCAAT-3′	5′-CCTTCGTGGGAGCCCTTT-3′
*JAM-A*	5′-GGTCAAGGTCAAGCTCAT-3′	5′-CTGAGTAAGGCAAATGCAG-3′
*ZO-1*	5′-CGCCAAATGCGGTTGATC-3′	5′-TTTACACCTTGCTTAGAGTCAGGGTTA-3′
*ZO-2*	5′-TCAAGCCAACAAGCTCAAAAAG-3′	5′-ATCGTTGGCTGAATTCACGTT-3′
βactin	TATCCACCTTCCAGCAGATGT	AGCTCAGTAACAGTCCGCCTA

### Analysis of gut microbiota

Feces collected from the mice were stored in a freezer at − 80 °C until DNA extraction. Genomic DNA was extracted using a NucleoSpin Microbial DNA Kit (Macherey–Nagel, Düren, Germany). Nine variable regions (V1–V9) in 16S rRNA provide the most useful information for phylogenetic and taxonomic studies. A two-step polymerase chain reaction (PCR) was performed on the purified DNA samples to obtain sequence libraries. The first PCR was performed to amplify and use a 16S (V3–V4) metagenomic library construction kit for NGS (Takara Bio Inc., Kusatsu, Japan) with the primer pairs 341F (5ʹ-TCGTCGGCAGCGTCAGATGTGTATAAGAGACAGCCTACGGGNGGCWGCAG-3ʹ) and 806R reverse primer (5ʹ-GTCTCGTGGGCTCGGAGATGTGTATAAGAGACAGGGACTACHVGGGTWTCTAAT-3ʹ) that corresponded to the V3–V4 region of the 16S rRNA gene.

The second PCR was performed to add the index sequences for the Illumina sequencer with a barcode sequence using the Nextera XT index kit (Illumina, San Diego, CA, USA). The prepared libraries were subjected to 250 paired-end base sequencing using the MiSeq Reagent v3 kit and MiSeq (Illumina) at the Biomedical Center of Takara Bio.

Quantitative Insights into Microbial Ecology 2 was used to analyze the sequence data. The DADA2 model was used to denoise the sequence reads, and the amplicon sequence variant (ASV) and representative sequences were determined. The ASVs were taxonomically assigned using a sklearn classifier against the GreenGenes database (13_8). Principal coordinate analysis was used to compare beta-diversity based on UniFrac distances, and permutational analysis of variance was used for statistical analysis of beta-diversity. From the obtained gut microbiota data, linear discriminant and effect size analyses were performed to identify the alternation in the abundance of the gut microbiota in mice after 1 week of Uro A administration.

### Induction of DSS colitis

Six-week-old male WT mice (C57BL/6) were used for the study. An experimental acute colitis model was induced using 2.5% DSS (molecular weight: 36,000–50,000 Da; lot no. M8667; MP Biomedicals, Santa Ana, CA, USA) in the drinking water for 7 days. The mice were euthanized under anesthesia precisely 7 days after the initiation of DSS treatment.

We evaluated colon length and the DAI, which was calculated by scoring changes in stool consistency, occult blood positivity, gross bleeding, and body weight, as previously described^45^. We used three grades of stool consistency (0, normal; 2, loose; and 4, diarrhea), three grades of occult blood (0, negative; 2, occult blood-positive; and 4, gross bleeding), and five grades of weight loss (0, no loss or weight gain; 1, 1–5% loss; 2, 5–10% loss; 3, 10–20% loss; and 4, > 20% loss). After evaluating the DAI, the mice were sacrificed, the entire colon was resected from the cecum to the anus, and colon length was measured as an indirect marker of intestinal inflammation. Distal colon specimens were preserved in 10% neutral buffered formalin for histological evaluation. Post-fixation, the specimens were embedded in paraffin, split into 7-µm sections, and subjected to hematoxylin and eosin staining.

### Induction of TNBS colitis

Colitis was induced in mice that were minimally anesthetized with ketamine/xylazine via a catheter by intrarectal administration of 200 mg/kg TNBS (Sigma–Aldrich Japan, Tokyo, Japan) dissolved in 30% ethanol^46,47^.

Mice in the control group were administered 30% ethanol. After 3 days post-TNBS administration, the mice were euthanized, and their colons were extracted for examination. The inflicted colonic damage was evaluated and ranked based on predetermined criteria. Macroscopic grading included visible impairments, serosal adhesions, diarrhea, strictures, and increased bowel wall thickness. All grading was executed by a singular individual under blinded conditions, thereby preventing observer bias.

### Cell culture

LS174T is a human colon adenocarcinoma cell line registered under the CL‑188TM mark at the American Type Culture Collection (Manassas, VA, USA). This cell line, which demonstrates traits of mucin-secreting intestinal epithelial cells, has been extensively used as a representative intestinal goblet cell line. Cultivation of LS174T cells spanned 1 week; it was performed using Dulbecco’s Modified Eagle Medium (DMEM) fortified with 2 mM l-glutamine, 10% heat-inactivated fetal bovine serum (FBS), and 100 U/mL penicillin. The incubation conditions comprised a temperature of 37 °C within a humidified atmosphere containing 5% carbon dioxide and 95% air. LS174T cells, at a concentration of 2.5 × 10^5^ cells/mL, were distributed in 6‑well plates in preparation for the subsequent ELISA assay. CH-223191, an AhR antagonist, was dissolved in 100% dimethyl sulfoxide (DMSO), and a 10-µM concentration was administered. Similarly, ML385, functioning as an Nrf2 inhibitor, was prepared by dissolving it in 100% DMSO to create a stock solution and subsequently diluted to a 10-µM concentration before application^48^.

### Treatment of LS174T cells with Uro A

LS174T cells were cultured in a 24-well-plate or μ-dish (35-mm) imaging dishes (ibidi GmbH, Martinsried, Germany) until the point of complete confluence. The cells were treated with DMEM without FBS for 12 h before Uro A treatment. In this experiment, Uro A was dissolved in DMSO at a concentration of 10 µM. LS174 cells were cultured, followed by exposure to 10 µM Uro A for 3 h to quantify the concentration of secreted MUC2. The amount of MUC2 protein present in the cell supernatant was determined using a Mucin ELISA kit, specifically the SEA705Hu model (Cloud-Clone Corp. Wuhan, China), according to the procedural guidelines provided by the manufacturer.

### Fluorescence microscopy

LS174T cellular specimens were cultivated on 35-mm μ-dishes intended (ibidi GmbH, Martinsried, Germany) and subsequently exposed to a concentration of 10 µM Uro A for 24 h. Following this, the cellular specimens were stabilized using 4% paraformaldehyde dissolved in PBS for 10 min, permeated with a PBS solution incorporating 0.1% Triton X-100 for 5 min, and subjected to a 24-h incubation period at a temperature of 37 ℃ with a primary antibody, specifically ab97386, targeting MUC2. In the subsequent stage, the cells were incubated with goat anti-rabbit Alexa 594, rhodamine-phalloidin, and Hoechst 33352 secondary antibodies, employed as staining agents for MUC2 and F-actin. Hoechst 33352 was used to stain nuclear chromatin. In the final step, cellular specimens were meticulously examined using the all-in-one fluorescence BZ-X810 laser scanning microscope (Keyence, Milton Keynes, UK). Fluorescence intensity was quantified using ImageJ software (National Institutes of Health, Bethesda, MD, USA).

### Statement of ethics

All experiments were performed in accordance with the ARRIVE guidelines 2.0 and the Guide for the Care and Use of Laboratory Animals (National Research Council, 8th edition, 2011) and approved by the Institutional Ethical Committee for Animal Experiments of the Kyoto Prefectural University of Medicine (Kyoto, Japan) under Assurance Number M 2020-113.

### Statistical analysis

All analyses were performed using the JMP PRO version 14.0.0 (SAS Institute Japan Ltd.). The trend test was performed using linear contrast. The evaluation of average tendencies, segmented based on customarily distributed continuous parameters, was performed using an analysis of variance. In normally distributed data, continuous variables are represented as means with an additional or subtractive standard deviation. For non-normally distributed data, the data were expressed as the median and the interquartile range (IQR) (25%, 75%).

### Supplementary Information

Supplementary Legends.

Supplementary Figure S1.

Supplementary Figure S2.

## Data Availability

The datasets used and analyzed in the current study can be acquired from the corresponding author upon reasonable request.

## References

[1] Espín JC, Larrosa M, García-Conesa MT, Tomás-Barberán F (2013). Biological significance of urolithins, the gut microbial ellagic Acid-derived metabolites: The evidence so far. Evid. Based Complement Alternat. Med..

[2] Smeriglio A, Barreca D, Bellocco E, Trombetta D (2017). Proanthocyanidins and hydrolysable tannins: Occurrence, dietary intake and pharmacological effects. Br. J. Pharmacol..

[3] Olthof MR, Hollman PC, Katan MB (2001). Chlorogenic acid and caffeic acid are absorbed in humans. J. Nutr..

[4] Manach C, Williamson G, Morand C, Scalbert A, Rémésy C (2005). Bioavailability and bioefficacy of polyphenols in humans. I. Review of 97 bioavailability studies. Am. J. Clin. Nutr..

[5] McDougall GJ, Stewart D (2005). The inhibitory effects of berry polyphenols on digestive enzymes. Biofactors..

[6] Priyadarsini KI, Khopde SM, Kumar SS, Mohan H (2002). Free radical studies of ellagic acid, a natural phenolic antioxidant. J. Agric. Food Chem..

[7] Yang J (2022). Pomegranate extract improves colitis in IL-10 knockout mice fed a high fat high sucrose diet. Mol. Nutr. Food Res..

[8] Larrosa M (2010). Anti-inflammatory properties of a pomegranate extract and its metabolite urolithin-A in a colitis rat model and the effect of colon inflammation on phenolic metabolism. J. Nutr. Biochem..

[9] Rupasinghe HPV, Parmar I, Neir SV (2019). Biotransformation of cranberry proanthocyanidins to probiotic metabolites by Lactobacillus rhamnosus enhances their anticancer activity in HepG2 cells in vitro. Oxid. Med. Cell Longev..

[10] Stevens JF, Maier CS (2016). The chemistry of gut microbial metabolism of polyphenols. Phytochem. Rev..

[11] Peron G (2021). Crosstalk among intestinal barrier, gut microbiota and serum metabolome after a polyphenol-rich diet in older subjects with "leaky gut": The MaPLE trial. Clin. Nutr..

[12] France MM, Turner JR (2017). The mucosal barrier at a glance. J. Cell Sci..

[13] Singh R (2019). Enhancement of the gut barrier integrity by a microbial metabolite through the Nrf2 pathway. Nat. Commun..

[14] Yasuda-Onozawa Y (2017). Rebamipide upregulates mucin secretion of intestinal goblet cells via Akt phosphorylation. Mol. Med. Rep..

[15] Johansson ME (2008). The inner of the two Muc2 mucin-dependent mucus layers in colon is devoid of bacteria. Proc. Natl. Acad. Sci. USA..

[16] Lang T, Hansson GC, Samuelsson T (2007). Gel-forming mucins appeared early in metazoan evolution. Proc. Natl. Acad. Sci. USA..

[17] Corfield AP (2015). Mucins: A biologically relevant glycan barrier in mucosal protection. Biochim. Biophys. Acta..

[18] Deplancke B, Gaskins HR (2001). Microbial modulation of innate defense: Goblet cells and the intestinal mucus layer. Am. J. Clin. Nutr..

[19] Elamin E, Masclee A, Troost F, Dekker J, Jonkers D (2014). Cytotoxicity and metabolic stress induced by acetaldehyde in human intestinal LS174T goblet-like cells. Am. J. Physiol. Gastrointest. Liver Physiol..

[20] Okumura R (2016). Lypd8 promotes the segregation of flagellated microbiota and colonic epithelia. Nature..

[21] Van der Sluis M (2006). Muc2-deficient mice spontaneously develop colitis, indicating that MUC2 is critical for colonic protection. Gastroenterology..

[22] Liso M (2020). A specific mutation in Muc2 determines early dysbiosis in colitis-prone winnie mice. Inflamm. Bowel Dis..

[23] Johansson ME (2014). Bacteria penetrate the normally impenetrable inner colon mucus layer in both murine colitis models and patients with ulcerative colitis. Gut..

[24] Elinav E (2011). NLRP6 inflammasome regulates colonic microbial ecology and risk for colitis. Cell..

[25] Yin J (2019). Aryl hydrocarbon receptor activation alleviates dextran sodium sulfate-induced colitis through enhancing the differentiation of goblet cells. Biochem. Biophys. Res. Commun..

[26] Liu L (2022). Intestinal stem cells damaged by deoxycholic acid via AHR pathway contributes to mucosal barrier dysfunction in high-fat feeding mice. Int. J. Mol. Sci..

[27] Wen ZS (2019). Low molecular seleno-aminopolysaccharides protect the intestinal mucosal barrier of rats under weaning stress. Int. J. Mol. Sci..

[28] Chassaing B (2015). Dietary emulsifiers impact the mouse gut microbiota promoting colitis and metabolic syndrome. Nature..

[29] Shinoda M (2010). Early-stage blocking of Notch signaling inhibits the depletion of goblet cells in dextran sodium sulfate-induced colitis in mice. J. Gastroenterol..

[30] Nishida A (2018). Gut microbiota in the pathogenesis of inflammatory bowel disease. Clin. J. Gastroenterol..

[31] Kettle H, Louis P, Holtrop G, Duncan SH, Flint HJ (2015). Modelling the emergent dynamics and major metabolites of the human colonic microbiota. Environ. Microbiol..

[32] Chen T (2017). Fiber-utilizing capacity varies in Prevotella- versus Bacteroides-dominated gut microbiota. Sci. Rep..

[33] Chambers ES (2015). Effects of targeted delivery of propionate to the human colon on appetite regulation, body weight maintenance and adiposity in overweight adults. Gut..

[34] Miyamoto J (2023). Host metabolic benefits of prebiotic exopolysaccharides produced by Leuconostoc mesenteroides. Gut Microbes..

[35] Kajiwara-Kubota M (2023). Partially hydrolyzed guar gum increased colonic mucus layer in mice via succinate-mediated MUC2 production. NPJ Sci. Food..

[36] Maslowski KM (2009). Regulation of inflammatory responses by gut microbiota and chemoattractant receptor GPR43. Nature..

[37] Smith PM (2013). The microbial metabolites, short-chain fatty acids, regulate colonic Treg cell homeostasis. Science..

[38] Ma S, Yeom J, Lim YH (2022). Specific activation of hypoxia-inducible factor-2α by propionate metabolism via a β-oxidation-like pathway stimulates MUC2 production in intestinal goblet cells. Biomed. Pharmacother..

[39] Kim SH (2006). Novel compound 2-methyl-2H-pyrazole-3-carboxylic acid (2-methyl-4-o-tolylazo-phenyl)-amide (CH-223191) prevents 2,3,7,8-TCDD-induced toxicity by antagonizing the aryl hydrocarbon receptor. Mol. Pharmacol..

[40] Ohkawa H, Ohishi N, Yagi K (1979). Assay for lipid peroxides in animal tissues by thiobarbituric acid reaction. Anal. Biochem..

[41] Huang W (2012). HMGB1 increases permeability of the endothelial cell monolayer via RAGE and Src family tyrosine kinase pathways. Inflammation..

[42] Vijay-Kumar M (2007). Deletion of TLR5 results in spontaneous colitis in mice. J. Clin. Invest..

[43] Costantini TW (2010). Quantitative assessment of intestinal injury using a novel in vivo, near-infrared imaging technique. Mol. Imaging..

[44] Gerkins C, Hajjar R, Oliero M, Santos MM (2022). Assessment of gut barrier integrity in mice using fluorescein-isothiocyanate-labeled dextran. J. Vis. Exp..

[45] Cooper HS, Murthy SN, Shah RS, Sedergran DJ (1993). Clinicopathologic study of dextran sulfate sodium experimental murine colitis. Lab. Invest..

[46] McCafferty DM (1999). Role of inducible nitric oxide synthase in trinitrobenzene sulphonic acid-induced colitis in mice. Gut..

[47] Sugimoto N (2008). Points of control exerted along the macrophage-endothelial cell-polymorphonuclear neutrophil axis by pecam-1 in the innate immune response of acute colonic inflammation. J. Immunol..

[48] Liu X (2018). Isoliquiritigenin ameliorates acute pancreatitis in mice via inhibition of oxidative stress and modulation of the Nrf2/HO-1 pathway. Oxid. Med. Cell Longev..

