# Supplementary material for: Urolithin A-mediated augmentation of intestinal barrier function through elevated secretory mucin synthesis
Source: Sci Rep. 2024 Jul 8;14:15706. doi: 10.1038/s41598-024-65791-x (PMC11231190; doi:10.1038/s41598-024-65791-x)
Supplement: Supplementary file 1 — Supplementary Legends. [file 41598_2024_65791_MOESM1_ESM.docx]

**Figure S1. MUC2 mRNA levels in the colonic mucosal epithelium**

The mRNA expression of MUC2 in the colon epithelium shows that the MUC2 levels of control group, 20 mg/kg Uro A group, and 100 mg/kg Uro A group gradually increased, although no significant difference was observed between these groups (control group (n=6); 3.3×10^-3^±3.8×10^-4^, 20 mg/kg Uro A group (n=6); 4.0×10^-3^±4.6×10^-4^, 100 mg/kg Uro A group (n=6); 4.8×10^-3^±5.7×10^-4^, control group versus 20 mg/kg Uro A group; p=0.370, and control group versus 100 mg/kg Uro A group; p=0.165)

Uro A, urolithin A;

**Figure S2. Schematic diagram of the induction of the TNBS colitis mouse model**

(a) Schematic diagram of the induction of the TNBS injury mouse model.

(b) Representative macroscopic findings in the colon on day 3 after the induction of TNBS injury.

(c) Effects of Uro A administration on the colonic macroscopic score. The macroscopic score was evaluated as described in the Materials and Methods section. The macroscopic score decreased in the Uro A group after 1 week of DSS administration (control group (n=6): 3.8±1.1, Uro A group (n=7): 2.7±1.3, p=0.077, Wilcoxon rank sum test)

TNBS, trinitrobenzene sulfonic acid
