# Supplementary figures and images for: Urolithin A-mediated augmentation of intestinal barrier function through elevated secretory mucin synthesis
Source: Sci Rep. 2024 Jul 8;14:15706. doi: 10.1038/s41598-024-65791-x (PMC11231190; doi:10.1038/s41598-024-65791-x)

**Figure S1**

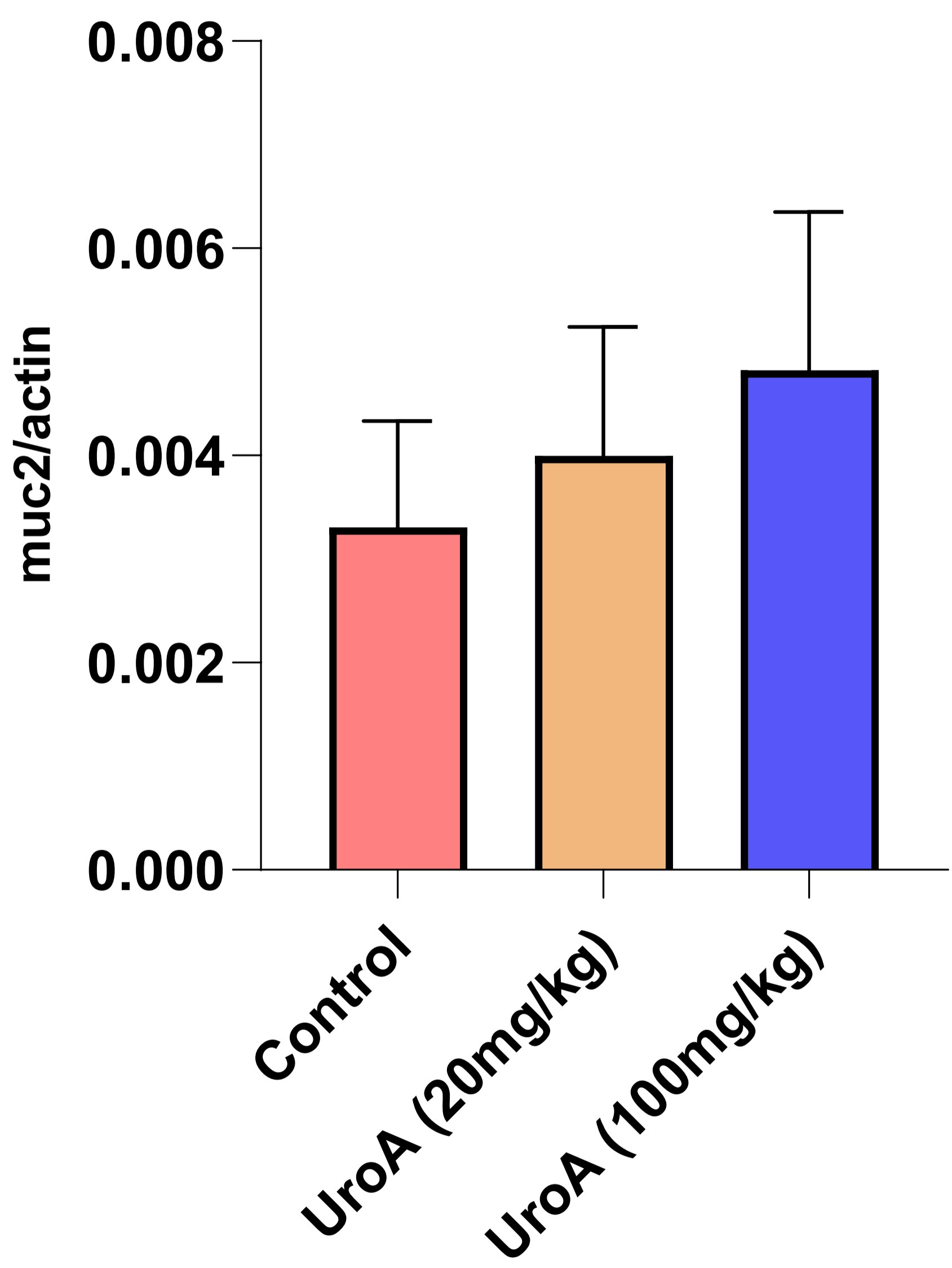

Supplement: Supplementary file 2 — Supplementary Figure S1. [file 41598_2024_65791_MOESM2_ESM.pdf]

Figure S2

(a)

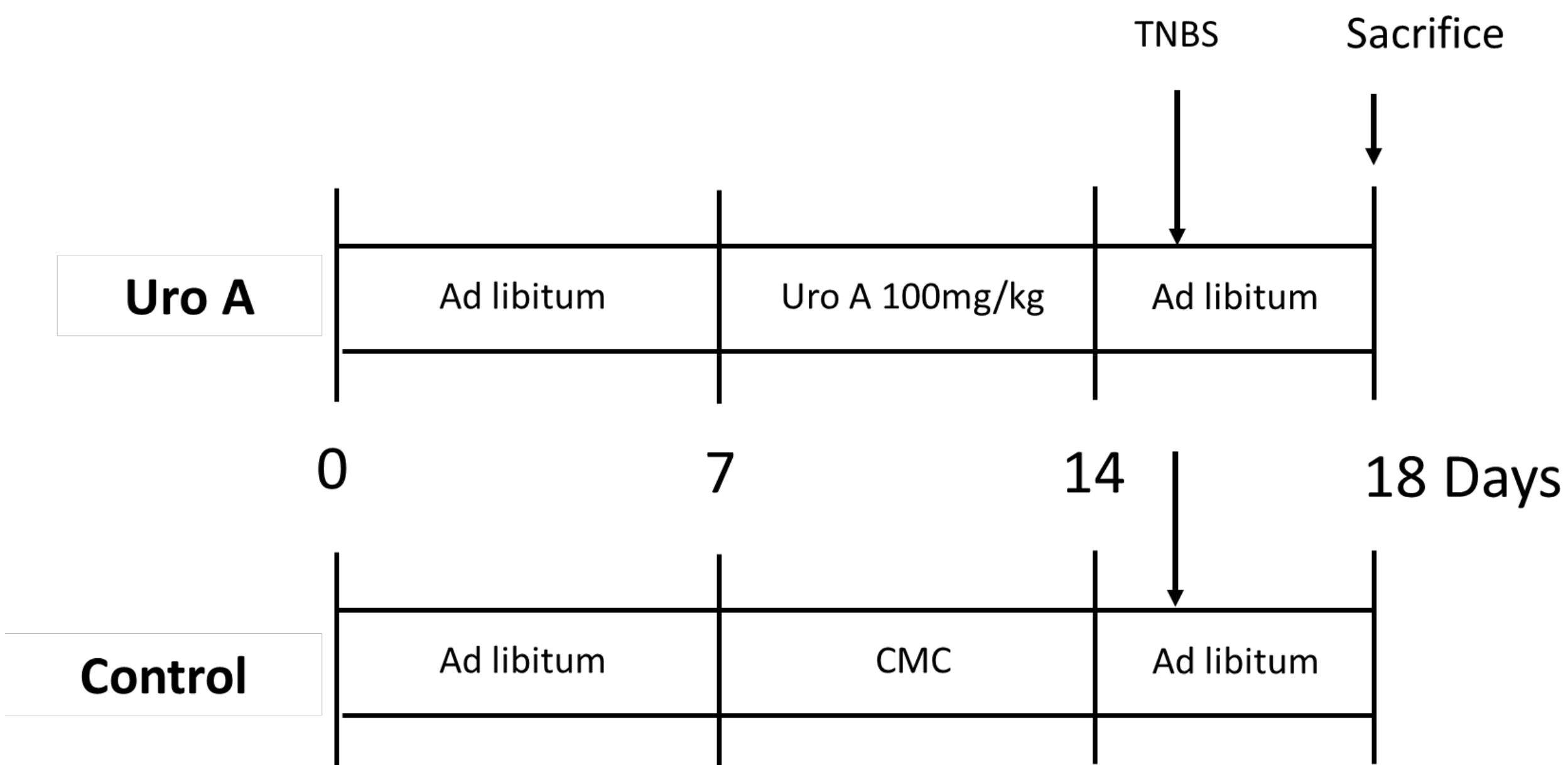

(b)

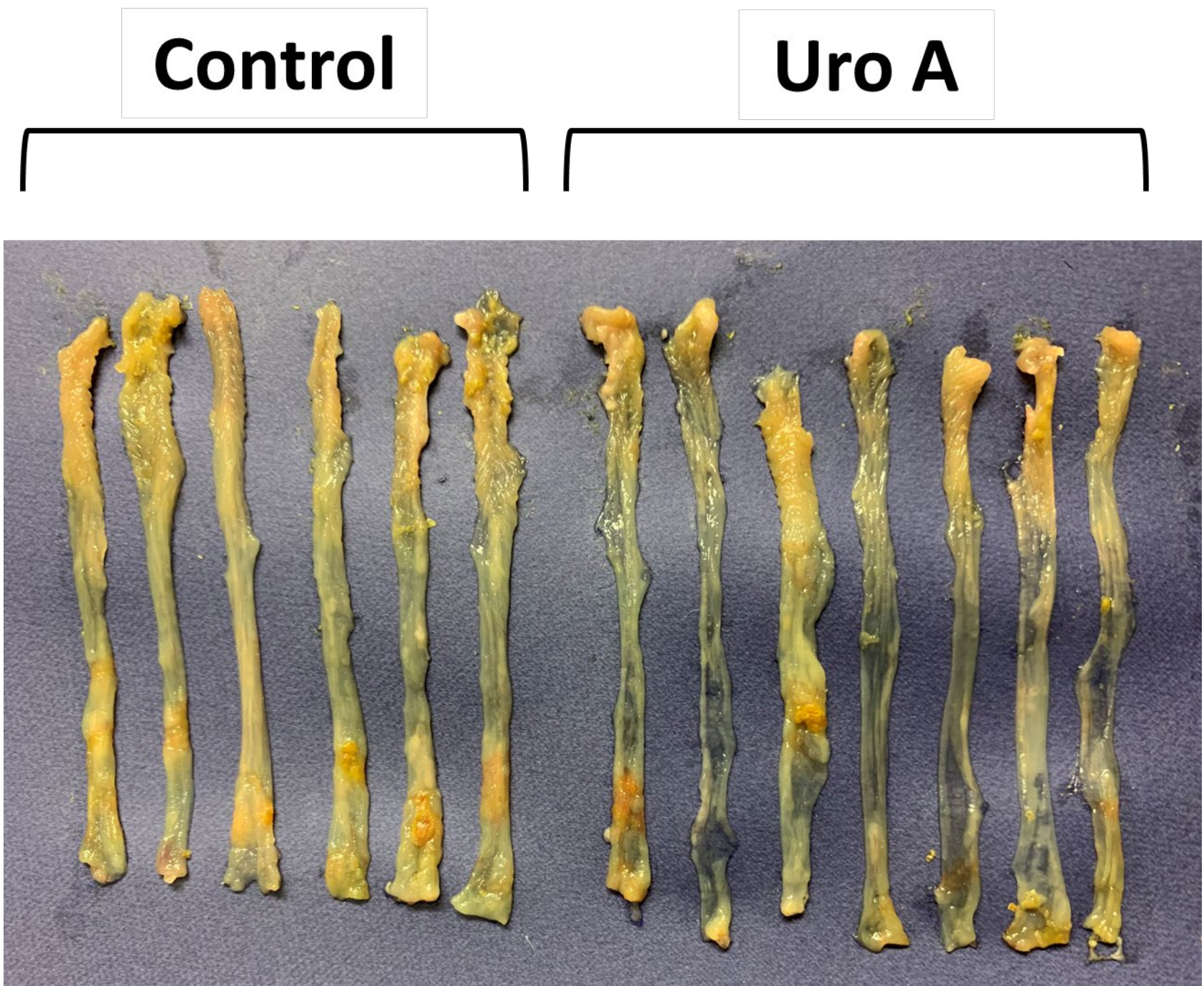

(c)

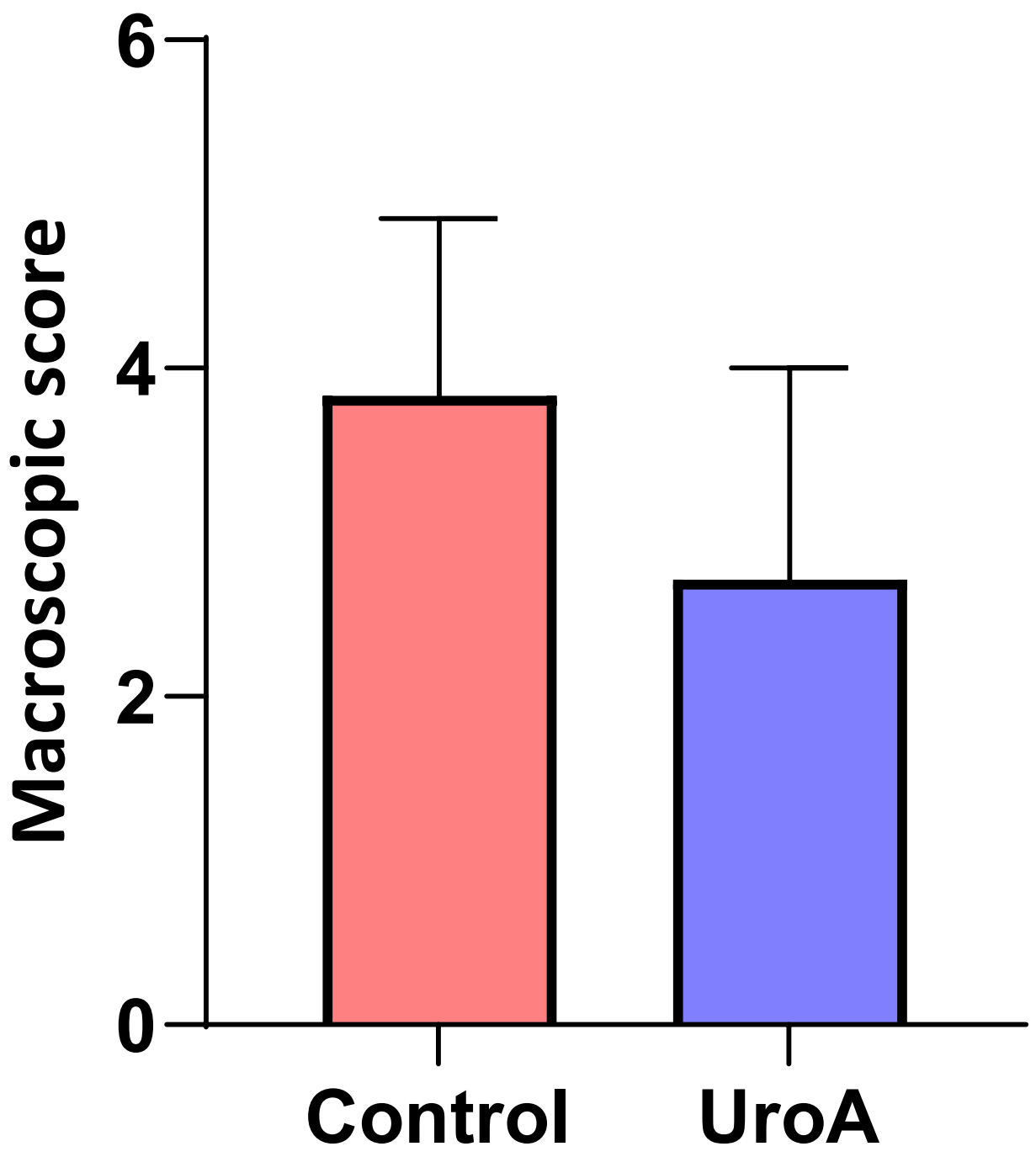

$p = 0.077$

Supplement: Supplementary file 3 — Supplementary Figure S2. [file 41598_2024_65791_MOESM3_ESM.pdf]
